# The combined Hf and Nd isotope evolution of the depleted mantle requires Hadean continental formation

**DOI:** 10.1126/sciadv.ade2711

**Published:** 2023-03-24

**Authors:** Meng Guo, Jun Korenaga

**Affiliations:** Department of Earth and Planetary Sciences, Yale University, P.O. Box 208109, New Haven, CT, USA.

## Abstract

The onset and rates of continental growth are first-order indicators of early Earth dynamics, and whether substantial crust existed in the Hadean or much later has long been debated. Here, we present a theoretical analysis of published Hf and Nd isotopic data representing the depleted mantle and demonstrate that continental growth must have started in the early Hadean. Whereas the traditional interpretation of depleted mantle signatures in crustal rocks assumes unrealistic instantaneous mantle mixing, our modeling incorporates the effect of a finite mixing time over which these signatures are recorded in rocks produced through mantle melting. This effect is shown to delay, by as much as 0.65 to 0.75 billion years, the appearance of the earliest depleted mantle signatures in continental crust. Our results suggest that published observations of εHf, ε^143^Nd, and μ^142^Nd require Hadean growth of continental crust, with a minimum of 50% of today’s continental volume already existing by the end of Hadean.

## Graphical abstract

**Figure:**
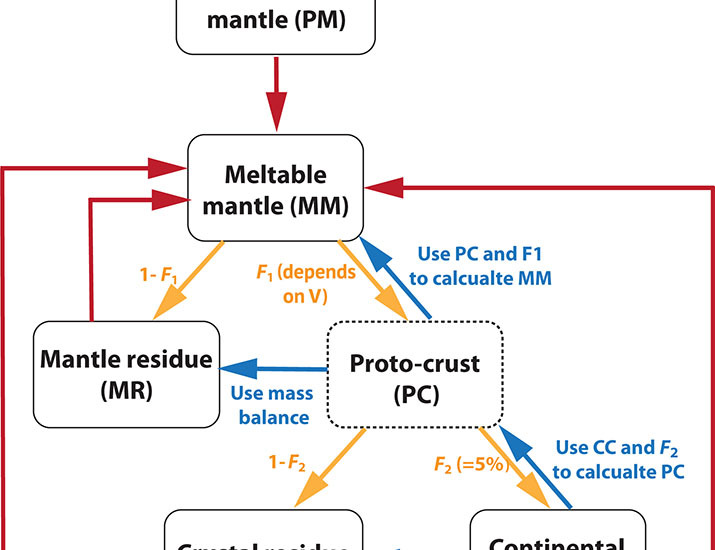

## Teaser

Inclusion of mantle mixing timescales into crustal growth models pushes crustal growth back into the early Hadean.

## INTRODUCTION

The formation of continental crust plays a central role in stabilizing climate [e.g., (*1*)] and providing unique environments for the development of life [e.g., (*2*)]. As the generation of continental crust is commonly associated with plate tectonics (*3*), the onset of continental formation can potentially constrain the timing and mechanisms of solid Earth differentiation as well as the style of early mantle convection [e.g., (*4*)]. However, given the scarcity of the geological data from the early Earth due to crustal reworking and destruction, the growth history of continental crust remains highly controversial, ranging from rapid extraction soon after Earth formation [e.g., (*5*–*7*)] to late gradual growth after ~3.8 billion years (Ga) ago [e.g., (*8*–*10*)].

A variety of methods have been applied to study the history of continental growth. The most direct observations come from the isotope signatures of the depleted mantle recorded in mantle-derived basalts [e.g., (*7*, *9*, *11*–*13*)], although indirect measurements such as the evolution of atmospheric composition can also provide some constraints [e.g., (*14*)]. The extraction of the continental crust is reflected in the geochemical evolution of the depleted mantle, and isotope systems in basalts such as samarium-neodymium (Sm-Nd) [e.g., (*12*, *15*)] and lutetium-hafnium (Lu-Hf) [e.g., (*16*)] can provide important constraints on this reservoir. The daughter isotopes in these systems are more incompatible during mantle melting and subsequent differentiation to continental crust than the parent isotopes, which results in lower parent-daughter ratios in the continental crust and higher ratios in the depleted mantle relative to the chondritic uniform reservoir (CHUR) or the terrestrial standard. With the continuous extraction and recycling of continental crust, the isotopic signature of the depleted mantle evolves accordingly, which can then be used to track crustal evolution.

Geochemical box modeling has been used to investigate the isotopic evolution of the depleted mantle to quantify the history of continental growth [e.g., (*5*, *7*, *9*, *11*, *17*)]. This approach is based on the complementary nature between the continental crust and the depleted mantle, and it can, in principle, constrain the net growth of continental crust in the most direct manner (*4*). Despite its long-standing popularity, there are two major deficiencies in existing box models. First, the geochemical data of mantle-derived rocks, which are the key observations constraining box modeling, have traditionally been interpreted as an immediate result of mantle depletion [e.g., (*7*, *8*, *18*)]. However, during mantle melting and crustal differentiation, the signature of crustal extraction is carried by a newly created highly depleted mantle residue. This depleted residue is unlikely to remelt unless it is heated even more or is well mixed with surrounding, more fertile components [e.g., (*19*–*21*)]. The need for remixing, which does not take place instantly, effectively delays the appearance of depleted mantle signatures in observable crustal rocks. Hereinafter, this phenomenon is referred to as “the effect of finite-time mixing on mantle melting”. The traditional interpretation of depleted mantle signals implicitly assumes instantaneous mixing and thus underestimates the timing of mantle depletion and crustal extraction. Second, geochemical box models tend to use only geochemical constraints, but other observations, e.g., from a geophysical or geological perspective, can provide additional information on crust-mantle differentiation. For example, by assuming different histories of continental recycling, DePaolo (*11*) and Armstrong (*5*) proposed contrasting patterns of continental growth using the same Nd isotope observations, with the former supporting the gradual growth of continental crust and the latter suggesting rapid growth. The evolving rate of crustal recycling could be incorporated with geochemical box modeling and be tested against the present-day distribution of crustal formation age (*14*). In addition, the initial depth of mantle melting (*14*) and the rate of mantle convection should be consistent with the thermal evolution of the mantle (*4*). From the perspective of solid Earth system, a preferred crustal evolution model should be consistent with not only geochemical observations but also geophysical and geological constraints. Whereas box modeling provides a quantitative basis for studies of crustal evolution, therefore, an improved theoretical framework is required to better assimilate our current understanding of mantle melting and solid Earth evolution.

In this study, we improve on traditional box modeling by quantitatively exploring the effect of finite-time mantle mixing on mantle melting. In addition, in our new model, crustal growth is simulated along with the thermal evolution of the mantle, and these outputs are checked against the present-day distribution of continental crust formation ages (*22*) and the evolution of mantle potential temperature (*23*), respectively. The thermal evolution of mantle controls the changing rate of plate velocity, which is tied to the mantle stirring rate (*24*). With the continuous generation and recycling of the continental crust, depleted and enriched heterogeneities are introduced to the mantle, and the former is consumed after being well mixed with the more fertile surroundings. The level of mantle heterogeneity is thus controlled by the evolution of continental crust and the rate of mantle mixing. In our model, the extent of mantle heterogeneity is evaluated by random sampling of the meltable components [e.g., (*25*–*27*)], and the results are compared to geochemical observations. By doing so, we estimate a possible range for the onset of the generation of the depleted mantle and complementary continental formation in addition to the mantle mixing rate in early Earth. Our model is the first to incorporate finite-time mixing effect on mantle melting, which serves as a more complete theoretical framework for chemical differentiation within the solid Earth.

A range of geochemical constraints can be considered in our modeling approach, and in this study, we use the combined observations of ε^143^Nd, μ^142^Nd, and εHf, because each of them has a unique advantage in tracking the history of continental growth. The ε [= 10^4^(*R*_sample_ − *R*_CHUR_)/*R*_CHUR_] and μ [= 10^6^(*R*_sample_ − *R*_standard_)/*R*_standard_] notations represent the difference between the daughter-stable isotope ratio in a sample (*R*_sample_) and the reference reservoir (*R*_CHUR_ or *R*_standard_), with positive and negative values suggesting being more depleted and more enriched, respectively, compared to the reference reservoir. The evolution of ε^143^Nd in the depleted mantle has been used to infer the history of continental growth since the 1980s [e.g., (*7*, *11*, *12*, *15*)]. The long half-life of ^147^Sm-^143^Nd (147 Ga) makes ε^143^Nd a good tracer for the long-term evolution of crust-mantle differentiation. The existence of the continental crust and the depleted mantle since the end of Hadean (~4.0 Ga ago) was proposed based on the large range of both strongly negative and positive ε^143^Nd in the Eoarchean rocks of southern West Greenland [e.g., (*15*, *28*, *29*)]. However, given that post-crystallization events could modify the ε^143^Nd signatures, the exact scale of the early mantle depletion has been challenged [e.g., (*18*, *30*, *31*)].

On the other hand, μ^142^Nd is considered to be more faithful to early differentiation events. With the half-life of 103 Ma, ^146^Sm became quickly extinct after ~0.5 Ga, so the ^146^Sm-^142^Nd isotope systematics are virtually unaffected by later alteration that can fractionate Sm and Nd. Because the post-crystallization events could still dilute the early ε^142^Nd signatures through mixing with isotopically distinct material, the preserved ε^142^Nd signatures should be considered as a minimum magnitude. The μ^142^Nd records display highly depleted mantle signatures in early Earth [e.g., (*31*, *32*)], which can be explained by either early crust-mantle differentiation [e.g., (*7*, *33*–*35*)] or inefficient mantle mixing of a heterogenous primitive mantle after magma ocean solidification [e.g., (*26*, *36*–*38*)]. These two different possibilities have never been evaluated together, but it is important to do so because neither of them can be theoretically ruled out. Our new theoretical framework allows us to explore these two possibilities jointly. Regardless of the origin of the early μ^142^Nd signals, the observed decreases of μ^142^Nd signals must reflect progressive mixing in the convecting mantle; therefore, the evolution of μ^142^Nd amplitude provides an important constraint on the stirring rate of the early mantle (*27*), even if the primitive mantle is not initially heterogeneous.

The behavior of the ^176^Lu-^176^Hf system parallels that of ^147^Sm-^143^Nd [e.g., (*16*)]. The shorter half-life of ^176^Lu (37.1 Ga) compared with that of ^147^Sm and the larger fractionation between Lu and Hf make it a more sensitive probe for crust-mantle differentiation. Moreover, zircon can provide εHf along with a robust crystallization age using the U-Pb geochronometer [e.g., (*6*, *10*, *39*, *40*)]. Fisher and Vervoort (*10*) investigated the εHf of Eoarchean zircons from southern West Greenland and suggested that there was no evidence for a depleted mantle before 3.8 Ga. Thus, in contrast to the notion of substantial continental crust and depleted mantle already existing at the end of Hadean arising from the interpretation of ε^143^Nd in Eoarchean rocks [e.g., (*15*, *28*, *29*)], the εHf of Eoarchean zircons seem to support much delayed crustal growth [e.g., (*16*, *41*–*43*)]. This is puzzling considering their similar geochemical behavior and their parallel signatures during the later part of Earth history [e.g., (*10*, *29*, *44*)].

Several mechanisms have been proposed to explain this apparent decoupling of εHf and ε^143^Nd in early Earth. First, the Sm-Nd and Lu-Hf isotope systems may be decoupled as a result of magma ocean solidification (*45*). Unique element partitioning in Ca-perovskite can lead to the formation of a primitive lower mantle with supra-chondritic Sm/Nd and chondritic Lu/Hf compositions. Second, a supra-chondritic Earth with respect to Sm/Nd has been proposed, requiring the existence of an unsampled complementary subchondritic reservoir in deep Earth (*36*). However, later studies by Burkhardt *et al.* (*46*) and Bouvier and Boyet (*47*) suggest that the offset of ^142^Nd between Earth and chondrites likely reflects nucleosynthetic anomalies rather than early Earth differentiation. The bulk Earth Sm/Nd ratio thus remains chondritic in this model, with no need for a hidden reservoir. Third, Hoffmann *et al.* (*29*) propose that, in addition to the effect of magma ocean solidification, the observed decoupling may be partially explained by subduction, according to the selective addition of Nd from subducted components to arc-like basalts, which is observed in modern subduction settings. Last, the redistribution of Sm and Nd during orogenic events (through post-crystallization tectonothermal events) may compromise the original Nd isotopic composition of ancient rocks (*18*). To summarize, the early decoupling of εHf and ε^143^Nd may have resulted from metamorphic disturbances or early differentiation processes in the silicate Earth. However, these mechanisms have never been quantitatively investigated by theoretical studies of continental growth. We have built a new box model to simultaneously explore Nd and Hf isotopic constraints on continental growth. We tackle the issue of εHf and ε^143^Nd decoupling by exploring scenarios of a chemically homogeneous primitive mantle to a chemically heterogeneous one and by quantifying how our inference on continental growth is affected by this difference in the initial conditions.

In what follows, we first present a brief description of our modeling framework, then summarize the results, and discuss how the combined εHf, ε^143^Nd, and μ^142^Nd evolution constrains the growth of continental crust. A full description of our method is provided in the Methods.

## RESULTS

**Fig. 1.**
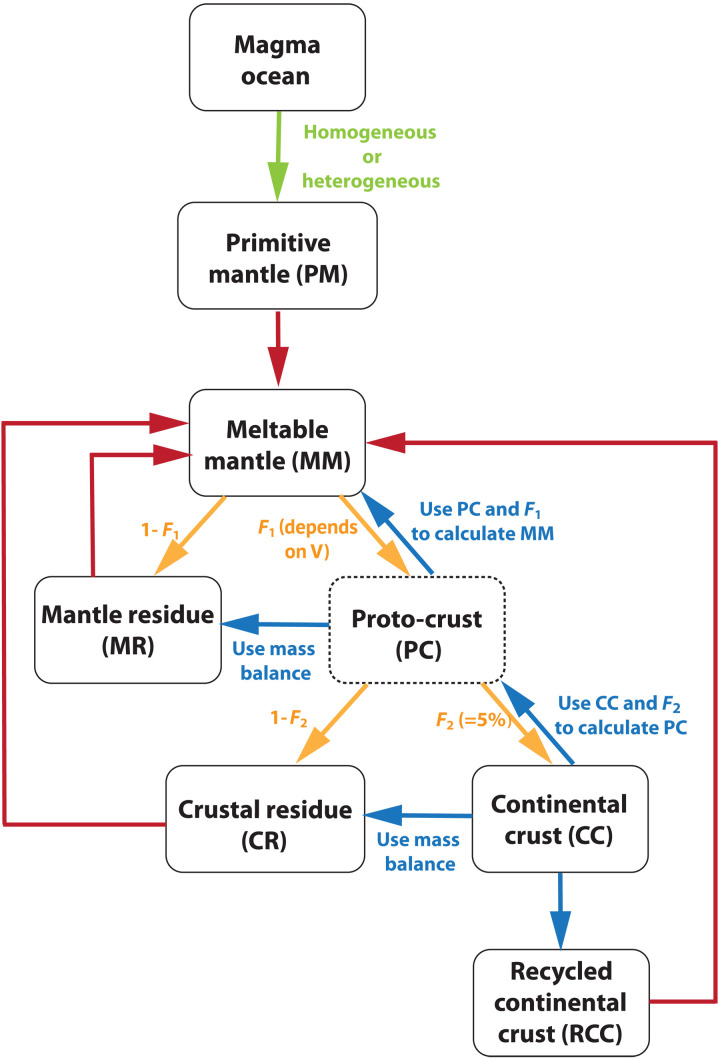
Model structure for the coupled chemical evolution of mantle and continental crust. The solid boxes represent the silicate reservoirs, and the dashed box labeled “proto-crust” is consumed during the generation of continental crust. The green arrow represents the solidification of magma ocean into a homogenous or heterogenous primitive mantle. The red arrows denote the contribution of silicate reservoirs during mantle melting. The yellow and blue arrows indicate the mass balance calculation. At every time step, the generation of a new batch of continental crust produces new segments of mantle residue, crustal residue, continental crust, and recycled continental crust, and our modeling tracks the fate of many thousands of segments in a self-consistent manner.

In our model, a variety of crustal growth patterns, with the onset ranging from 4.55 to 3.8 Ga ago, is prescribed to determine the rates of crustal generation and recycling through Earth history, and the corresponding depleted mantle components are calculated according to mass balance with the newly generated continental crust (Fig. 1). As we aim to test vastly different possibilities, a wide range is used for the onset time. Its upper boundary (4.55 Ga ago) is chosen according to the timing of Moon-forming giant impact (*48*, *49*), corresponding to the possibility that plate tectonics can initiate immediately after magma ocean solidification (*4*). The lower bound (3.8 Ga ago) is set to be the latest proposed time of continental formation according to the εHf records in Greenland zircon [e.g., (*10*, *41*–*43*, *50*)], as we are using εHf as one of the observational constraints. As shown in fig. S1, the assumed crustal growth models are consistent with the present-day mass of continental crust and the distribution of formation ages (*22*), whereas the corresponding thermal evolution of the mantle is constrained by the history of mantle potential temperature (*23*). As shown in Fig. 1, we assume that the continental crust is produced from the two-stage melting of the mantle. First, a proto-crust is generated from the mantle and leaves a depleted mantle residue, with a time-dependent melt fraction (*F*_1_) that varies with plate velocity. Here, the term “proto-crust” denotes basaltic products from a single-stage melting of the mantle, which can be oceanic crust, oceanic plateaus, seamount, large igneous provinces, or basaltic eruptions in the continental domain, which are generated continuously over the model run. Then, the proto-crust is assumed to differentiate immediately to form a continental crust to leave a crustal residue, with a melt fraction (*F*_2_) set to 5%. Meanwhile, a fraction of the continental mass is recycled into the mantle, and the evolution of continental recycling is checked against the present-day distribution of crustal formation age (*22*). As a result, the degree of mantle heterogeneity increases with the continuous generation and recycling of the continental crust. Each heterogeneity in the mantle is continuously stretched by convection [e.g., (*25*–*27*)], and this effect of finite-time convective mixing is emulated by extending the length scale of each heterogeneity (and thus reducing the thickness thereof) with time. The rate of mantle mixing is controlled by the evolution of plate velocity (see Methods). We simultaneously track the evolution of εHf, ε^143^Nd, and μ^142^Nd in the depleted mantle and monitor both the mantle average composition and the extent of heterogeneity, the latter of which is evaluated by random sampling of the meltable components in the mantle (*25*). The distribution coefficients of elements are calculated according to their present-day concentrations in the bulk silicate Earth (BSE) (*51*), the continental crust (*52*), and the average melting fraction during net crustal generation (*F*) using the approach of Hofmann (see Methods) (*53*). This approach of calculating distribution coefficients aims to encapsulate all the complex processes required to produce continental crust (*9*). The detailed description of our method is provided in Methods.

To assess the influence of early decoupling between ε^143^Nd and εHf on our modeled onset of continental formation, we compare the chemically homogeneous and heterogeneous cases for the initial primitive mantle. As mentioned in Introduction, one possible explanation for the discrepancy between εHf and ε^143^Nd signatures in early Earth is that the preservation of ε^143^Nd data is subject to greater post-crystallization alterations compared to εHf (*10*), which implies that there is no clear signature of mantle depletion before 3.8 Ga ago. We use a homogenous mantle, with the chondritic Sm/Nd and Lu/Hf ratios, to simulate this scenario. On the other hand, the discrepancy can also be explained by a heterogenous primitive mantle with suprachondritic Sm/Nd and chondritic Lu/Hf mantle components after magma ocean solidification (*45*). A recent study of magma ocean solidification also suggests that an early mantle may be chemically heterogeneous, characterized with small-scale Fe-rich blobs embedded in a depleted mantle matrix (*54*). The unique partitioning behavior of Ca-perovskite fractionates Sm/Nd and Lu/Hf in a contrasting manner between the upper and lower primitive mantle (*45*). To explore this scenario, we use a heterogeneous primitive mantle, with the depleted Sm/Nd and chondritic εHf signals in the lower mantle. These initial chemical heterogeneities would gradually be erased by convective mixing, and εHf is expected to couple with ε^143^Nd in the later part of Earth history.

**Fig. 2.**
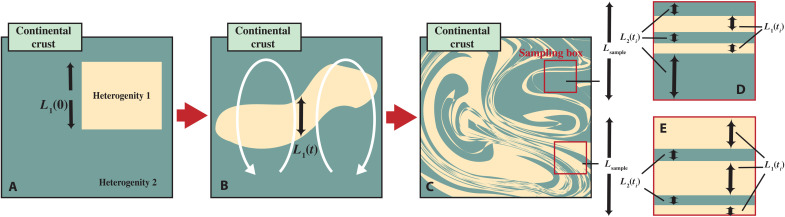
Schematic illustration of finite-time mixing in the mantle. (**A** to **C**) Effect of finite-time mixing on mantle heterogeneities in the convecting mantle and (**D** and **E**) random sampling in the mantle. In our geochemical box modeling, we emulate this mixing process by extending the length scale of each heterogeneity (and thus reducing the thickness thereof) in a way consistent with the thermal evolution of the mantle.

The most important feature of our model is that it takes into account the effect of finite-time mixing on mantle melting. The depleted or cold components, e.g., depleted mantle residue, crustal residue, and recycled continental crust, are unlikely to melt before being well mixed and brought up to shallow depths. To quantify a melting criterion in the mantle, we calculate the mantle stretching rate and overturn time according to the evolution of plate velocity, which is constrained by the observations of mantle potential temperature (*23*). Furthermore, the stretching rate can also be affected by volatile contents, with depleted and thus dry materials being more resistant to mantle stirring [e.g., (*55*, *56*)]. As shown in Fig. 2 (A to C), we assume that each heterogeneity enters the mantle with an initial width *L*(0), and the width *L*(*t*) decreases exponentially with time according to the stretching rate. This parameterization emulates the effect of an initially bulky heterogeneity being stretched into thin laminar in the mantle and mixed with the surroundings. A depleted or cold component can only contribute to mantle melting after one overturn time and has *L*(*t*) smaller than a critical width. As a result, the first appearance of the depleted mantle signal generated by continental extraction is determined by the rate of mantle mixing. On the other hand, the primitive mantle components can always melt, but we still need to track the evolution of their length scales to determine the degree of heterogeneity in the mantle.

The degree of mantle heterogeneity is assessed by random sampling in the convecting mantle. As illustrated in Fig. 2 (C to E), a sampling box of width *L*_sample_ is randomly placed in the mantle to represent a melting event, and meltable components are randomly picked to fill the sampling box according to their masses. A selected component occupies a part of *L*_sample_ according to its width *L*(*t*) (Fig. 2, D and E), and selection is repeated until the box is filled. The chemical composition of sampling box represents one mantle-derived rock at this time step. We take 300 random sampling results every 0.25 Ga, which we deem as sufficient to compare with the observations. The random sampling results are compared to the observations of ε^143^Nd, μ^142^Nd, and εHf in mantle-derived rocks.

**Fig. 3.**
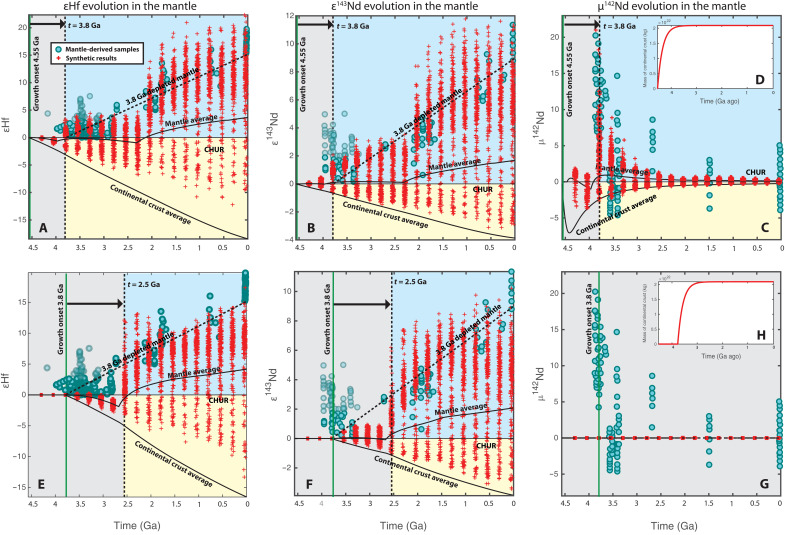
The evolution of εHf, ε^143^Nd, and μ^142^Nd in the depleted mantle during continental formation, with a homogenous post-magma-ocean primitive mantle. (**A** to **C** and **E** to **G**) Isotopic evolutions in mantle with crustal growth onset at (**D**) 4.55 Ga and (**H**) 3.8 Ga, respectively. The observations of εHf, ε^143^Nd, and μ^142^Nd evolution are shown in blue dots [(*10*, *15*, *16*, *29*, *31*–*35*, *37*, *38*, *39*, *48*, *70*–*83*) and references therein], and our modeling results are in red crosses. As the early strongly positive ε^143^Nd signals may be subject to post-crystallization events [e.g., (*18*, *30*, *31*)] and the strongly positive εHf signals exist only in detrital zircons but not in magmatic zircons (*10*), we use a lighter shade for these observations. The vertical green lines mark the onset of continental growth. The vertical dashed lines represent the timing of the earliest mantle depleted signals. The dashed lines labeled “3.8 Ga depleted mantle” represents the hypothetical evolution of depleted mantle when continental crust was extracted at 3.8 Ga. The modeled isotopic evolutions of the average mantle and continental crust are also shown in solid curves. The blue and yellow backgrounds denote the domains in which our modeling results show strongly positive and negative isotopic signals, respectively, whereas the gray background denotes the domain with no notable signals.

For the case of a homogenous primitive mantle, the synthetic mantle evolution of εHf, ε^143^Nd, and μ^142^Nd is shown in Fig. 3, with the onset of crustal growth at 4.55 Ga ago (Fig. 3, A to D) and 3.8 Ga ago (Fig. 3, E to H), respectively. In this scenario, the depleted mantle signatures are generated solely by crustal growth, because both the upper and lower parts of the primitive mantle have the isotopic composition of BSE. As a result, the timing of the earliest mantle depletion is determined by the onset of continental growth and the mixing rate of mantle convection.

Two conclusions may be drawn by comparing the results with the early and late onsets (Fig. 3). First, by incorporating the effect of finite-time mixing on mantle melting, the early depleted εHf and ε^143^Nd signals, which are generated by 4.55 Ga ago of crustal growth, can fit the hypothetical depleted mantle evolution starting at 3.8 Ga ago (Fig. 3, A and B). On the other hand, the onset of depleted εHf and ε^143^Nd signals in the case of crustal growth starting at 3.8 Ga ago is delayed to ~2.5 Ga ago (Fig. 3, E and F). This comparison suggests that the appearance of the depleted mantle signals at 3.8 Ga ago [e.g., (*10*)] requires crustal growth to start much earlier than that. In other words, the onset of depleted mantle signals should be considered as a lower bound on the onset of crustal growth. Second, in the case of a homogenous primitive mantle, the μ^142^Nd data require the onset of continental formation to be in the early Hadean (*7*, *33*–*35*). ^146^Sm quickly became extinct after ~0.5 Ga; therefore, the depleted mantle signature of μ^142^Nd can only be generated before this time through continental extraction (Fig. 3C). For comparison, when crustal generation starts at 3.8 Ga ago, μ^142^Nd cannot record mantle depletion signatures (Fig. 3G).

As explained in the Introduction, the observed decreases of μ^142^Nd signals through early Earth suggest that the mantle gradually becomes homogenized regarding μ^142^Nd [e.g., (*27*)]. Therefore, the appearance of the early positive μ^142^Nd signatures can constrain the rate of mantle mixing in the Hadean through the early Archean. On the basis of this, Hyung and Jacobsen (*27*) proposed that the decreasing trend of early μ^142^Nd signals was consistent with a mantle stirring time of 400 Ma since the early Hadean, assuming two isotopically distinct reservoirs (one enriched and one depleted). However, μ^142^Nd can also be also affected by the generation of continental crust and its recycling, and this is why we need to model both mantle mixing and continental growth simultaneously to constrain the rate of mantle mixing using the μ^142^Nd evolution. With the mantle mixing rate constrained, the amplitude of the depleted mantle signals is controlled by the onset of crustal growth and the distribution coefficients. We adjust the distribution coefficients within their uncertainties and search for the possible range of continental growth onset that can explain all three isotope systems. The results suggest that the beginning of continental growth should be earlier than 4.45 Ga ago when the primitive mantle is chemically homogenous (fig. S2). When crustal generation starts later than 4.45 Ga ago, the large amplitude of μ^142^Nd in the early Archean requires that Nd to be considerably more incompatible than Sm, leading to the overshooting of ε^143^Nd signals (fig. S2).

**Fig. 4.**
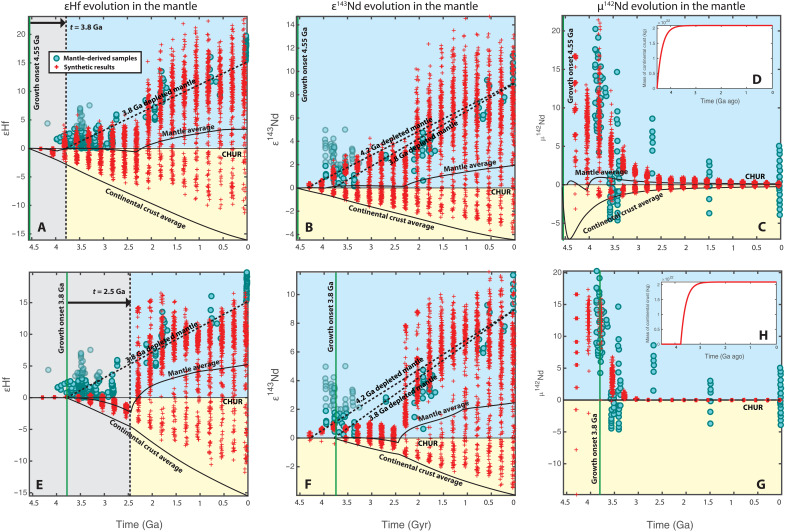
Same as but for the case of a heterogeneous post-magma-ocean primitive mantle. **Fig. 3** (**A** to **C** and **E** to **G**) The isotopic evolutions in the mantle with crustal growth onset at (**D**) 4.55 Ga and (**H**) 3.8 Ga, respectively.

A heterogenous primitive mantle (Fig. 4) results in depleted ε^143^Nd and μ^142^Nd signatures from the beginning (*45*), which is independent from the production of continental crust. Thus, the onset of continental generation is best seen from εHf evolution, whereas the evolutions of ε^143^Nd and μ^142^Nd are the results of both magma ocean solidification and continental formation. This can explain the decoupling of the early εHf and ε^143^Nd signals [e.g., (*10*)]. On the other hand, similar to the conclusion for the homogenous mantle case, the earliest depleted εHf signal generated by the continental growth starting at 4.55 Ga ago can fit the hypothetical depleted mantle evolution starting at 3.8 Ga ago (Fig. 4A), and the εHf produced by 3.8 Ga ago of continental crust generation is again delayed to ~2.5 Ga ago (Fig. 3E). The extents of delay are similar for both the homogenous and heterogenous mantle cases because the mantle mixing rates required by early μ^142^Nd signatures are similar.

With the mantle mixing rate being constrained by μ^142^Nd, we adjust the distribution coefficients to search for the latest possible onset of continental growth that is compatible with all three isotope systems. The results suggest that the beginning of continental growth should be again no later than 4.45 Ga ago (fig. S3). In this scenario, however, the early μ^142^Nd and ε^143^Nd signatures come mostly from the heterogenous primitive mantle; thus, they do not provide robust constraints on the onset of continental growth. The most important constraint comes from εHf, and the 4.45 Ga ago onset is the latest time for the earliest depleted εHf signal to appear around 3.8 Ga ago.

## DISCUSSION

Our results suggest that the combined observations of εHf, ε^143^Nd, and μ^142^Nd require the early Hadean growth of continental crust, regardless of the initial heterogeneity of the primitive mantle. The initial chemical state of the post-magma ocean mantle can potentially provide important constraints on the earliest phase of mantle convection (*57*). Previous box models often assume a homogenous mantle after magma ocean solidification [e.g., (*7*, *9*, *11*, *17*)], although rare examples of models with a heterogenous mantle exist [e.g., (*26*, *27*)]. However, because the isotopic signatures of the early depleted mantle can be generated by both crust-mantle differentiation [e.g., (*7*, *33*–*35*)] and magma ocean solidification [e.g., (*36*, *38*, *45*, *58*)], it is vital to investigate both. Rosas and Korenaga (*7*) focused on the implication of a homogeneous primitive mantle and suggested a rapid early growth of continental crust because the generation of early strongly positive μ^142^Nd has to come from continental growth in the Hadean. On the other hand, Hyung and Jacobsen (*27*) assumed an initially heterogeneous post-magma ocean mantle, which could produce early depleted mantle signals of μ^142^Nd. As a result, they suggested that there was no need for continental growth to have occurred in the Hadean, although their main conclusion is the Hadean onset of plate tectonics. In our model, we quantitatively test these two explanations of early depleted mantle signatures, and both point to the early Hadean growth of continental crust. Even with a heterogenous initial mantle, early continental generation is required by the εHf data if the effect of finite-time mixing is considered.

As described in Results and Methods, our box modeling incorporates the effect of finite-time mixing on mantle melting. The results suggest that the appearance of the earliest depleted mantle signatures generated by continental formation may be delayed for ~0.7 Ga. Therefore, an appearance of the depleted mantle signals at 3.8 Ga ago does not indicate the Archean onset of continental growth; it actually requires the Hadean continental growth. The extent of this delay is controlled by the efficiency of convective mixing in the mantle. In our model, we calculate the stretching factor of mantle heterogeneities according to an evolving plate velocity, under the assumption of a logarithmic mixing regime in the mantle (see Methods) (*24*). Similar parameterization has been adopted previously [e.g., (*27*)], and our time-dependent parameterization results in fast mixing in the Hadean and followed by a slower mixing rate during the rest of Earth history. In our model, the stretching factor also varies among different mantle heterogeneities according to their volatile contents, or viscosity, with the depleted mantle residues being less stretched during convection [e.g., (*55*, *56*)]. The results of using a uniform stretching factor among all components are shown in fig. S4, which display poorer fit with observations in the later part of Earth history. As explained in Introduction, the effect of convective mixing in early Earth is reflected in the evolution of μ^142^Nd amplitude. As a result, although the current understanding of sub-solidus mantle convection is still incomplete [e.g., (*59*)], we can use the observed decreases of μ^142^Nd signals to quantify the “effective” mixing rate of actual mantle convection in early Earth. Because the onset of depleted mantle signatures is controlled by both the efficiency of mantle mixing and the start of continental formation, with the former being constrained by μ^142^Nd, we are able to estimate the earliest possible continental generation to be within 0.1 Ga after magma ocean solidification.

Other than constraining the early mantle mixing rate, the variation of μ^142^Nd data also indicates the extent of mantle heterogeneities. The large range of the early μ^142^Nd observations reflects the nonuniform depletion of the mantle [e.g., (*27*, *53*)]. Many previous box models tried to fit the highly depleted μ^142^Nd (or equivalently, ε^143^Nd) data with the average composition of the depleted mantle, and one common solution is to preserve 20 to 80% of the primitive mantle [e.g., (*7*, *9*, *11*)]. Whereas this approach may be justified as a way to simulate the evolution of the most depleted end-member, the preservation of such a large fraction of the primitive mantle, if taken at face value, conflicts with important seismological observations that favor whole-mantle convection [e.g., (*60*, *61*)]. In our model, we do not sequester any part of the mantle from convective mixing. As seen in our results (Figs. 3 and 4), the depleted mantle can naturally display large variations in isotopic signatures, while its average composition is only moderately depleted. Our modeling approach thus helps to reconcile geochemical and geophysical observations.

The amplitude of the earliest depleted mantle signals indicates the scale of continental formation. As explained in Results, the amplitude is controlled by both the rate of crustal generation and the differences between the distribution coefficients of parent and daughter isotopes. We follow the approach of Hofmann (*53*) to calculate the possible range of bulk distribution coefficients, which allows our model to reproduce the element concentrations in the modern continental crust. By varying the bulk distribution coefficients, both gradual and rapid growth of continental crust can result in similar isotopic signatures in the depleted mantle, with a minimum of 80% crust already existing by the end of Hadean for an initially homogenous primitive mantle, whereas 50% for a heterogenous one (fig. S5). This result supports the notion that a large amount of continental crust already existed on Earth at 4.4 Ga ago [e.g., (*5*, *6*, *14*, *62*)]. The continuous creation of a considerable amount of continental crust in early Earth is more consistent with the early initiation of plate tectonics than other non-plate tectonic regimes [e.g., (*63*)], which has important applications for planetary habitability [e.g., (*1*)] and the origin of life [e.g., (*64*)].

## METHODS

We calculate the evolving chemical and isotopic compositions of the depleted mantle and the continental crust during solid Earth differentiation. We first model continental growth to constrain the mass transfer rates between the mantle and the crust, and then we simulate the corresponding thermal evolution of the mantle to determine mantle stirring rate and overturn time. The extent of isotopic heterogeneities in the mantle is tracked using random sampling.

### The evolution of the continental crust

We follow Rosas and Korenaga (*7*) for the parameterization of crustal growth and recycling rates

$$M_{\mathrm{cc}}(t)=\frac{M_{\mathrm{cc}}(t_{p})}{1-e^{-\kappa_{g}(t_{p}-t_{s})}}[1-e^{-\kappa_{g}(t-t_{s})}]$$ (1)

$$K_{\mathrm{rc}}(t)=R_{s}+\frac{R_{p}-R_{s}}{1-e^{-\kappa_{r}(t_{p}-t_{s})}}[1-e^{-\kappa_{r}(t-t_{s})}]$$ (2)

$$\frac{dM_{\mathrm{cc}}(t)}{dt}=K_{\mathrm{cc}}(t)-K_{\mathrm{rc}}(t)$$ (3)

where *M*_cc_(*t*) is the mass of continental crust at time *t*, *t*_s_ is the onset of crustal formation, and *t*_p_ represents the present day. Thus, *M*_cc_(*t*_p_) is the present-day mass of continental crust (2.09 × 10^22^ kg). The terms κ_g_ and κ_r_ are the decay constants for the continental generation rate, *K*_cc_, and the recycling rate, *K*_rc_, respectively, and *R*_s_ and *R*_p_ are the rates of crustal recycling at *t*_s_ and *t*_p_, respectively. This parameterization allows us to simulate nearly all the proposed continental growth models, except for a larger volume (or mass) of continents in the past [e.g., (*65*)]. We note that a considerably greater volume of continental crust in the past would lead to an unrealistic surface environment (*4*). The continental crust is unlikely to have been much thicker in the past (*66*, *67*); thus, a larger continental volume means a greater coverage of Earth’s surface. However, a planet with its surface mostly covered by continents would not support the operation of plate tectonics, which is essential for the generation and recycling of continental crust (*4*).

Following Guo and Korenaga (*14*), the formation age distribution of continental crust is denoted by *m*(*t*, τ), where *t* is time and τ is formation age. The summation of the *m*(*t*, τ) over time τ gives the total crustal mass at time *t*

$$M_{\mathrm{CC}}(t)=\int_{0}^{t}m(t,\tau )d\tau$$ (4)

In our model, recycling uniformly affects the continental parts that are formed at different times. Thus, the evolution of *m*(*t*, τ) with age-independent recycling is modeled as

$$\frac{\partial m(t,\tau )}{\partial t}=K_{mc}(t)\delta (t-\tau )-\frac{K_{\mathrm{rc}}(t)}{M_{\mathrm{CC}}(t)}m(t,\tau )$$ (5)

where δ(*t*) is the Dirac delta function. The present-day cumulative formation age distribution, CFD(τ), can be calculated as

$$\mathrm{CFD}(\tau )=\frac{1}{M_{\mathrm{CC}}(t_{p})}\int_{0}^{\tau }m(t_{p},\tau^{\prime })d\tau^{\prime }$$ (6)

In our model, different continental growth patterns are simulated by varying parameters in Eqs. 1 to 3, and their corresponding cumulative formation age distribution is checked against Korenaga (*22*).

### The thermal evolution of the mantle

The thermal evolution of the mantle controls the melting depth beneath mid-ocean ridges and plate velocity, which determine, respectively, the melting fraction and the stirring rate in mantle. The evolution of mantle potential temperature, *T*_p_, can be tracked backward in time according to global energy balance

$$C_{m}\frac{dT_{p}(t)}{dt}=H(t)-Q(t)+Q_{c}(t)$$ (7)

where *C*_m_ is the heat capacity of the mantle (4.97 × 10^27^ J/K), *H* is the mantle heat production, *Q* is the mantle heat flux, and *Q*_c_ is the core heat flux. Our approach is almost the same as that of Guo and Korenaga (*14*), with a modification for the Hadean. To be self-contained, the approach of Guo and Korenaga (*14*) is described first in the following.

To solve for *T*_p_, we first model *Q*_c_ to be changing linearly with time as follows

$$Q_{c}(t)=\text{Δ}Q_{c}(t_{p}-t)/t_{p}+Q_{c}(t_{p})$$ (8)

where the present-day core heat flux *Q*_c_(*t*_p_) is considered to vary between 5 to 15 TW. The term Δ*Q*_c_ represents the difference between initial and present-day core heat flux, which varies between 2 to 5 TW.

Then, we track the evolution of *H* using the decay constants and the heat production rates of major heat producing isotopes within solid Earth

$$H(t)=H(t_{p})\frac{\sum_{i=1}^{4}c_{i}p_{i}e^{\lambda_{i}t}}{\sum_{i=1}^{4}c_{i}p_{i}}$$ (9)

where *i* varies between 1 to 4, representing the heat producing isotopes ^238^U, ^235^U, ^232^Th, and ^40^K; c*_i_* and p*_i_* are the present-day relative concentration and the heat generation rate of the isotope in interest, respectively; λ*_i_* is the decay constant; and *H*(*t*_p_) is the present-day mantle heat production, which is calculated as the total BSE heat production of 16 ± 3 TW (*51*) minus the present-day continental crust heat production, *H*_CC_(*t*_p_)

$$H(t_{p})=(16+3\varepsilon_{1})-H_{\mathrm{CC}}(t_{p})$$ (10)

and the present-day continental crust heat production is considered to be (*52*)

$$H_{\mathrm{CC}}(t_{p})=7.5+2.5\varepsilon_{2}$$ (11)

where ε_1_ and ε_2_ are independent random variables that vary between −1 and 1.

Last, *Q* is considered to be constant (36 TW) throughout Earth history following Korenaga (*68*). Thus, the evolution of mantle potential temperature *T*_p_ can be calculated by integrating Eq. 7 backward in time.

Knowing the evolution of *T*_p_, the initial depth of mantle melting, *Z*(*t*), is considered to be

$$Z(t)=\frac{T_{p}(t)-1150}{\;{g\rho }_{m}[1.2\times 10^{-7}-{\left(dT/dP\right)}_{S}]}$$ (12)

where *g* is the gravitational acceleration (9.8 m/s^2^), ρ_m_ is the density of mantle (3300 kg/m^3^), and (*dT*/*dP*)*_S_* is the mantle adiabatic gradient (1.54 × 10^−8^ K/Pa).

The temporal evolution of plate velocity, *V*, can be calculated using its relationship with *Q* and *T*_p_

$$V(t)=V(t_{p}){\left[\frac{Q(t)}{Q(t_{p})}\frac{T_{p}(t_{p})}{T_{p}(t)}\right]}^{2}$$ (13)

where the present-day plate velocity *V*(*t*_p_) is 5 cm/year and the present-day mantle heat flux *Q*(*t*_p_) is the difference between the present-day total terrestrial heat flux (46 ± 3 TW) and *H*_CC_(*t*_p_)

$$Q(t_{p})=(46+3\varepsilon_{3})-H_{\mathrm{CC}}(t_{p})$$ (14)

where ε_3_ is a random variable, which can vary between −1 and 1.

Following Miyazaki and Korenaga (*57*), we assume that the tempo of early Hadean plate tectonics was considerably faster than present day. Such rapid plate tectonics is possible if magma ocean solidification results in a wet, chemically heterogeneous mantle, and hence, the tempo of plate tectonics is expected to decrease as convective mixing gradually erases chemical heterogeneities. Thus, we adjust the Hadean plate velocity to be 10 times higher at the beginning of Earth history and then linearly decrease to the Archean plate velocity expected in Eq. 13 (fig. S1F). The effect of this modification in plate velocity on thermal evolution is negligible because of its limited duration.

### Magma Ocean crystallization

Our preparation of the heterogenous primitive mantle is based on, but is more general than, the approach of Caro *et al.* (*45*). It is noted that the results of Caro *et al.* (*45*) require a specific mass fraction of Ca-perovskite and a melt fraction in the lower mantle [for details, see figure 3 in (*45*)] and that their equation 9 in their Supplementary Material is incorrect. We model a heterogenous primitive mantle considering that a magma ocean crystalizes from the bottom into lower depleted and upper enriched portions, which can provide the strongly positive and negative μ^142^Nd signals observed in the early Archean. Following Caro *et al.* (*45*), the upper mantle constitutes 22 to 30% of the mass of the whole mantle and has a composition of 57% olivine, 14% garnet, and 29% clinopyroxene, whereas the lower mantle forms the remaining part with 16% ferropericlase, 79 to 75% Mg-perovskite, and 5 to 9% Ca-perovskite.

The chemical structure of the lower and upper mantle after crystallization are determined by calculating first the element bulk distribution coefficients, then the average ratios of ^147^Sm/^144^Nd and ^176^Lu/^177^Hf, and lastly the element concentrations in the upper and lower mantle. The bulk distribution coefficients of Sm, Nd, Lu, and Hf are calculated by using the following (*45*)

$$D=\sum_{i=1}^{3}D_{i}f_{i}$$ (15)

where *D_i_* and *f_i_* are the partition coefficient and mass fraction of lower mantle minerals.

Second, the ratios of ^147^Sm/^144^Nd and ^176^Lu/^177^Hf in the lower and upper mantle are calculated according to the above bulk distribution coefficients and the mass fraction of lower mantle. For this part of the calculation, we use the following equations for equilibrium crystallization

$$R_{\mathrm{UM}}=R_{\mathrm{CHUR}}\frac{x(D^{d}-1)+1}{x(D^{p}-1)+1}$$ (16)

$$R_{\mathrm{LM}}=R_{\mathrm{CHUR}}\frac{D^{p}}{D^{d}}\;\frac{x(D^{d}-1)+1}{x(D^{p}-1)+1}$$ (17)

and for fractional crystallization

$$R_{\mathrm{UM}}=R_{\mathrm{CHUR}}\;\frac{(1-x{)}^{D^{p}-1}}{(1-x{)}^{D^{d}-1}}$$ (18)

$$R_{\mathrm{LM}}=R_{\mathrm{CHUR}}\frac{1-{\left(1-x\right)}^{D^{p}}}{1-{\left(1-x\right)}^{D^{d}}}$$ (19)

where *R*_CHUR_, *R*_UM_, and *R*_LM_ are the initial ratios of parent to daughter isotope in the BSE, the upper mantle, and the lower mantle, respectively; *D*^p^ and *D*^d^ are the bulk distribution coefficients of parent and daughter isotopes, respectively; and *x* is defined as the mass fraction of lower mantle, which can be expressed as

$$x=\frac{M_{\mathrm{LM}}}{M_{\mathrm{BSE}}}$$ (20)

Then, the element concentrations in the upper and lower mantle (*C*_UM_ and *C*_LM_) can be obtained as follows for equilibrium crystallization

$$\frac{C_{\mathrm{BSE}}}{(1-x)-xD}$$ (21)

$$C_{\mathrm{LM}}=DC_{\mathrm{UM}}=\frac{DC_{\mathrm{BSE}}}{(1-x)-xD}$$ (22)

and for fractional crystallization

$$C_{\mathrm{BSE}}{\left(1-x\right)}^{\left(D-1\right)}$$ (23)

$$C_{\mathrm{LM}}=DC_{\mathrm{UM}}=DC_{\mathrm{BSE}}{\left(1-x\right)}^{\left(D-1\right)}$$ (24)

To reproduce the ^147^Sm/^144^Nd and ^176^Lu/^177^Hf signatures in the Hadean depleted mantle after magma ocean solidification (*45*), the amount of Ca-perovskite in the lower primitive mantle is set to be 5%, with a melt fraction of 30% for equilibrium crystallization and 23% for fractional crystallization; similar isotope signatures can be obtained by either mode of crystallization by adjusting the melt fraction. Our results shown in Results are based on equilibrium crystallization.

### Finite-time mixing in the mantle

As explained in Introduction, we consider that the cold or depleted mantle heterogeneities cannot participate in mantle melting until experienced one mantle overturn time (*t*_overturn_) and have a width below a critical width (*l*_critical_). This melting criteria simulates the finite-time mixing effect on mantle melting.

The *t*_overturn_ is determined by mantle depth, *h* (2900 km), and the average plate velocity, $\bar{V}$, as

$$t_{\mathrm{overturn}}=\frac{2h}{\bar{V}}$$ (25)

The calculated *t*_overturn_ is ~110 Ma. The *l*_critical_ in our model is set to be 15 km to facilitate the simulation of evolving chemical heterogeneities. A smaller *l*_critical_ results in more delayed and homogenized depleted mantle signals, which requires even earlier onset of continental formation under the same mantle stirring rate, whereas a larger *l*_critical_ results in *t*_overturn_ solely controlling the remelting of mantle heterogeneities.

The width of each mantle heterogeneity decreases with time during mantle mixing as

$$L(t)=\frac{L(0)}{\alpha {\left(t\right)}^{t}}$$ (26)

where *L*(0) is the initial width of mantle material of interest and α is the stretching factor. As a consequence, mantle heterogeneity increases in length and becomes more distributed within the mantle. We consider the *L*(0) of mantle residue, crustal residue, and recycled continental crust to be the melting depth of mantle *Z* (Eq. 12), 100 km, and 5 km, respectively [e.g., (*27*)].

Under the assumption of a logarithmic mixing regime in the mantle (*24*), which is associated with pure shear and has a cumulative strain exponentially increasing with time, the stretching factor α can be expressed as

$$\alpha (t)=\exp[\dot{\varepsilon }(t)dt]$$ (27)

where *dt* is the time step used in our box modeling (=1 Ma) and $\dot{\varepsilon }$ is the strain rate of mantle (*24*)

$$\dot{\varepsilon }(t)=0.08\;f\;V(t)/h$$ (28)

where the factor 0.08 is from Olson *et al.*, (*24*) and *f* is an additional scaling factor, which is set to 2 (except for the case of a homogeneous primitive mantle and a late onset of continental growth, for which *f* is set to 3) for primitive mantle, crustal residue, and recycled continental crust, to account for likely differences between two-dimensional isoviscous convection used in Olson *et al.*, (*24*) and actual mantle convection. For mantle residue, *f* is varied between 0.5 (Hadean) and 1.9 (post-Hadean) to simulate a slower stretching rate for more viscous materials [e.g., (*55*, *56*)]. Lower *f* for the Hadean is chosen to reflect a greater viscosity contrast expected from a wet Hadean mantle (*57*). The values of these scaling factors were determined to optimize the fit between model prediction and observation for all of the three isotope systems considered. These scaling factors allow us to emulate the effective mixing efficiency of actual three-dimensional mantle convection.

Following Kellogg *et al.* (*25*), the mantle mixing time may be defined as an *e*-folding time scale of stretching, i.e., $\tau =1/\dot{\varepsilon }(t)$. The average mixing time scale during the Hadean is ~90 Ma for primitive mantle, crustal residue, and recycled continental crust and ~360 Ma for mantle residue. From Archean to the present, the average mixing time scale is ~420 Ma for primitive mantle, crustal residue, and recycled continental crust and ~440 Ma for mantle residue. These mixing time scales are all shorter than the time lag between the onset of continental growth and the appearance of depleted signals (~700 Ma), demonstrating that calculating the mixing time scale alone is insufficient to quantify the potential time lag.

As mentioned in Introduction, the primitive mantle can always melt, so together with the mantle heterogeneities that satisfy the melting criteria, they compose the meltable mantle for the generation of continental crust. During the continuous continental growth and mantle mixing, the mass and composition of total meltable mantle evolves with time, which can be expressed as

$$M_{\mathrm{MM}}(t)=M_{\mathrm{LM}}(t)+M_{\mathrm{UM}}(t)+\sum M_{x}(t,\tau )$$ (29)

$$N_{\mathrm{MM}}(t)=N_{\mathrm{LM}}(t)+N_{\mathrm{UM}}(t)+\sum N_{x}(t,\tau )$$ (30)

$$C_{\mathrm{MM}}(t)=\frac{N_{\mathrm{MM}}(t)m}{N_{A}M_{\mathrm{MM}}(t)}$$ (31)

where *M*(*t*), *N*(*t*), and *C*(*t*) represent the mass, the number of atoms, and the concentration in mantle subreservoirs at time *t*, *m* is the element atomic mass, and *N*_A_ is the Avogadro’s number (6.022 × 10^23^). The subscripts LM, UM, and x denote lower primitive mantle, upper primitive mantle, and the meltable mantle heterogeneities (mantle residue, crustal residue, and recycled continental crust) that generated at time τ.

### Geochemical evolution in solid Earth during crust-mantle differentiation

Different formation histories of the continental crust and the thermal evolution of the mantle are prescribed to determine the geochemical differentiation of solid Earth. Assuming continuous two-stage melting of the mantle (Fig. 1) to create continental crust, the masses of mantle heterogeneities are calculated according to the mass balance with the newly generated continental crust (*M*_dCC_). First, the mass of a proto-crust can be calculated as

$$M_{\mathrm{PC}}(t)=\frac{M_{\mathrm{dCC}}(t)}{F_{2}}$$ (32)

where *F*_2_ is the melt fraction of basaltic material during the second-stage generation of continental crust, which is set to be 5% to match the present-day continental crust mass fraction of ~0.5% after two-stage melting from the mantle.

Then, according to the mass balance with the proto-crust, the corresponding mass of a crustal residue can be obtained as

$$M_{\mathrm{CR}}(t)=M_{\mathrm{PC}}(t)-M_{\mathrm{dCC}}(t)$$ (33)

whereas the mass of the total consumed meltable mantle, *M*_CMM_, during the generation of the proto-crust is

$$M_{\mathrm{CMM}}(t)=\frac{M_{\mathrm{PC}}(t)}{F_{1}(t)}$$ (34)

where *F*_1_ is the melt fraction during the first-stage generation of continental crust (i.e., the generation of oceanic crust). The evolution of *F*_1_ is controlled by the melting depth of mantle, *Z*, as

$$F_{1}(t)=\frac{Z(t)F_{1}(t_{p})}{Z(t_{p})}$$ (35)

where *F*_1_(*t*_p_) is the present-day melt fraction in the mantle (10%) and *Z* is calculated according to the thermal evolution of mantle (Eq. 12).

Knowing *M*_MM_ and *M*_PC_, the mass of a mantle residue during the first-stage mantle melting can be calculated as follows

$$M_{\mathrm{MR}}(t)=M_{\mathrm{CMM}}(t)-M_{\mathrm{PC}}(t)$$ (36)

The composition of each mantle heterogeneity is also tracked through time using the element bulk distribution coefficient, *D*. We calculate *D* of the target elements Sm, Nd, Lu, and Hf, following Hofmann (*53*)

$$D=\frac{C_{\mathrm{BSE}}(t_{p})-{FC}_{\mathrm{CC}}(t_{p})}{\left(1-F)C_{\mathrm{CC}}(t_{p}\right)}$$ (37)

where *C*_BSE_(*t*_p_) and *C*_CC_(*t*_p_) are the present-day element concentrations in the BSE and the continental crust, respectively and *F* is the average melting fraction during net crustal generation [0.9%; (*53*)]. In our model, *D*_Sm_, *D*_Nd_, *D*_Lu_, and *D*_Hf_ are set to be 0.0353, 0.0300, 0.1068, and 0.0684, respectively, for the scenario of rapid continental growth, whereas *D*_Nd_ and *D*_Lu_ are changed to 0.0288 and 0.1168, respectively, for gradual continental growth. These choices on distribution coefficients are made to minimize the differences between model predictions and observations.

During the first-stage of mantle melting, the proto-crust is generated from meltable mantle, whose composition can be calculated as follows

$$C_{\mathrm{PC}}(t)=\frac{C_{\mathrm{MM}}(t)}{F_{1}(t)+D-F_{1}(t)D}$$ (38)

$$N_{\mathrm{PC}}(t)=\frac{C_{\mathrm{PC}}(t)N_{A}M_{\mathrm{PC}}(t)}{m}$$ (39)

According to the mass balance with the proto-crust, the composition of the mantle residue can be calculated as following

$$N_{\mathrm{MR}}(t)=N_{\mathrm{CMM}}(t)-N_{\mathrm{PC}}(t)$$ (40)

$$C_{\mathrm{MR}}(t)=\frac{N_{\mathrm{MR}}(t)m}{N_{A}M_{\mathrm{MR}}(t)}$$ (41)

where the number of atoms of the isotope in interest in the consumed meltable mantle can be calculated as

$$N_{\mathrm{CMM}}(t)=\frac{C_{\mathrm{MM}}(t)N_{A}M_{\mathrm{CMM}}(t)}{m}$$ (42)

During the second-stage of mantle melting, the proto-crust melts to generate continental crust, whose composition can be calculated as

$$C_{\mathrm{dCC}}(t)=\frac{C_{\mathrm{PC}}(t)}{F_{2}+D-F_{2}D}$$ (43)

$$N_{\mathrm{dCC}}(t)=\frac{C_{\mathrm{dCC}}(t)N_{A}M_{\mathrm{dCC}}(t)}{m}$$ (44)

In our model, we assume equilibrium process for both stage of mantle melting. Similar results can be obtained for fractional crystallization by adjusting the difference between bulk partition coefficients of parent and daughter elements within their uncertainties.

According to mass balance with the newly generated continental crust, the composition of the crustal residue can be obtained using

$$N_{\mathrm{CR}}(t)=N_{\mathrm{PC}}(t)-N_{\mathrm{dCC}}(t)$$ (45)

$$C_{\mathrm{CR}}(t)=\frac{N_{\mathrm{CR}}(t)m}{N_{A}M_{\mathrm{CR}}(t)}$$ (46)

Last, the recycled continental crust has the same composition as the total crust.

Through Earth history, the evolution of ε^143^Nd, μ^142^Nd, and εHf systems are tracked within each reservoir as the following

$$\frac{d}{dt}N^{p}(t)=-\lambda N^{p}(t)$$ (47)

$$\frac{d}{dt}N^{d}(t)=\lambda N^{p}(t)$$ (48)

where *N*^p^ and *N*^d^ are the number of parent and daughter isotopes, respectively and λ is the decay constant.

### Random sampling in the mantle

We collect 300 random sampling results in the mantle every 0.25 Ga through Earth history to be compared with the observations of ε^143^Nd, μ^142^Nd, and εHf in mantle-derived rocks. Each random sampling represents one melting event in the mantle; thus, the sampling pool only includes the mantle components that fit with melting criteria, i.e., the meltable mantle. To fill the sampling box, we pick randomly from the pool of all meltable components, according to the following probability

$$P_{x}(t,\tau )=\frac{M_{x}(t,\tau )}{M_{\mathrm{MM}}(t)}$$ (49)

where *P*_x_(*t*, τ) and *M*_x_(*t*, τ) are the probability and mass at time *t* of a mantle component x that is generated at time τ and *M*_MM_(*t*) is the total mass of the meltable mantle at time *t*.

The selected mantle component usually occupies only a fraction of the sampling box, i.e., the width of the selected mantle component, *L*(*t*), is smaller than the width of the sampling box, *L*_sampling_ (Eq. 26 and Fig. 2, C and E). The width of the sampling box is set to be 100 km [e.g., (*25*)], which corresponds to the typical size of a melting region under mid-ocean ridges (*19*). We repeat this random selection process until the sampling box is completely filled

$$\sum_{i=1}^{n}L_{i}(t)=L_{\mathrm{sampling}}$$ (50)

where *i* runs through all the selected meltable components and *n* is the total number of selections needed to completely fill the sampling box. When the width of a selected component exceeds that of the sampling box, only part of the component is used to fill the box. The chemical composition of the sampling box is the weighted average of all the components within it.

The random sampling results of ε^143^Nd and μ^142^Nd are compared with the following observed Nd depleted mantle signatures: Bennett *et al.* (*15*), Morino *et al.* (*31*), Puchtel *et al.* (*32*), Rizo *et al.* (*34*), Roth *et al.* (*35*), Debaille *et al.* (*37*), Caro *et al.* (*38*), Bennett *et al.* (*69*), Baadsgaard *et al.* (*70*), Moorbath *et al.* (*71*), Blichert-Toft *et al.* (*72*), Murphy *et al.* (*73*), Jackson and Carlson (*74*), OʼNeil *et al.* (*75*), Kröner *et al.* (*76*), and references therein; whereas the results of εHf are compared with the following: Vervoort and Blichert-Toft (*16*), Hoffmann *et al.* (*29*), Fisher and Vervoort (*30*), Blichert-Toft *et al.* (*72*), Amelin *et al.* (*77*), Pietranik *et al.* (*78*), Zeh *et al.* (*79*), Zeh *et al.* (*80*), Nebel-Jacobsen *et al.* (*81*), Mueller and Wooden (*82*), Laurent and Zeh (*83*), Kröner *et al.* (*76*), and references therein. Considering that the early ε^143^Nd signals may be subjected to post-crystallization events [e.g., (*18*, *30*, *31*)] and that only detrital zircons, but no magmatic zircons, suggest early mantle depletion in terms of εHf (*10*), we use a lighter shade in Figs. 3 and 4 for the early strongly positive ε^143^Nd and εHf observations to show the level of confidence.
