# Supplementary material for: The combined Hf and Nd isotope evolution of the depleted mantle requires Hadean continental formation
Source: Sci Adv. 2023 Mar 24;9(12):eade2711. doi: 10.1126/sciadv.ade2711 (PMC10038339; doi:10.1126/sciadv.ade2711)
Supplement: Supplementary file 1 — Figs. S1 to S5 [file sciadv.ade2711_sm.pdf]

Science Advances
AAAS

Supplementary Materials for

The combined Hf and Nd isotope evolution of the depleted mantle requires Hadean continental formation

Meng Guo and Jun Korenaga

Corresponding author: Meng Guo, meng.guo@yale.edu

Sci. Adv. **9**, eade2711 (2023)
DOI: 10.1126/sciadv.ade2711

This PDF file includes:

Figs. S1 to S5

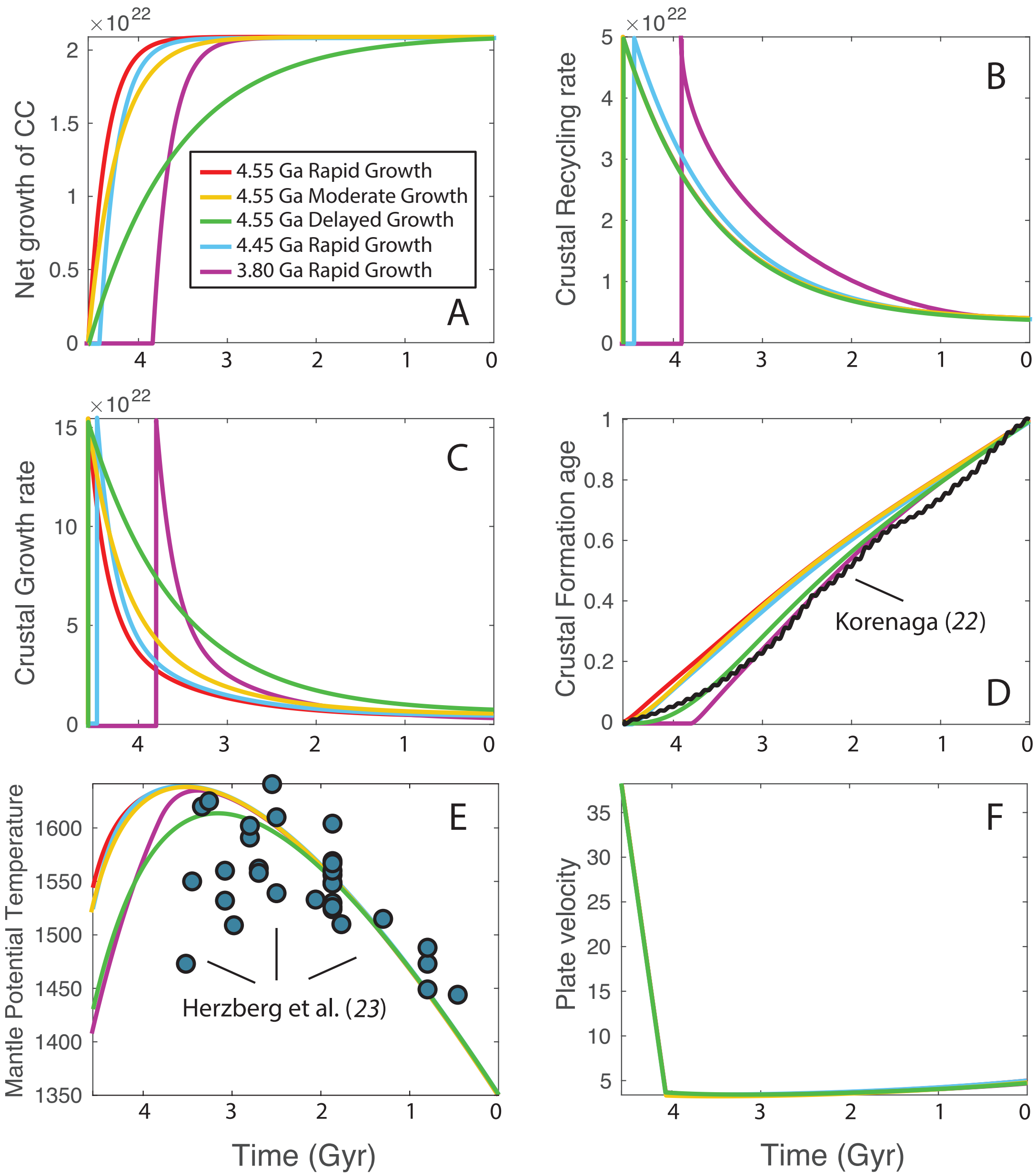

Fig. S1. The formation history of continental crust and the thermal evolution of the mantle. The evolution of **(A)** net growth of continental crust, **(B)** continental recycling rate, **(C)** continental generation rate, **(D)** crustal formation age distribution, **(E)** mantle potential temperature, and **(F)** plate velocity. The model synthetic evolutions are shown in colored lines, and the observations are shown in black line and dots (*22, 23*).

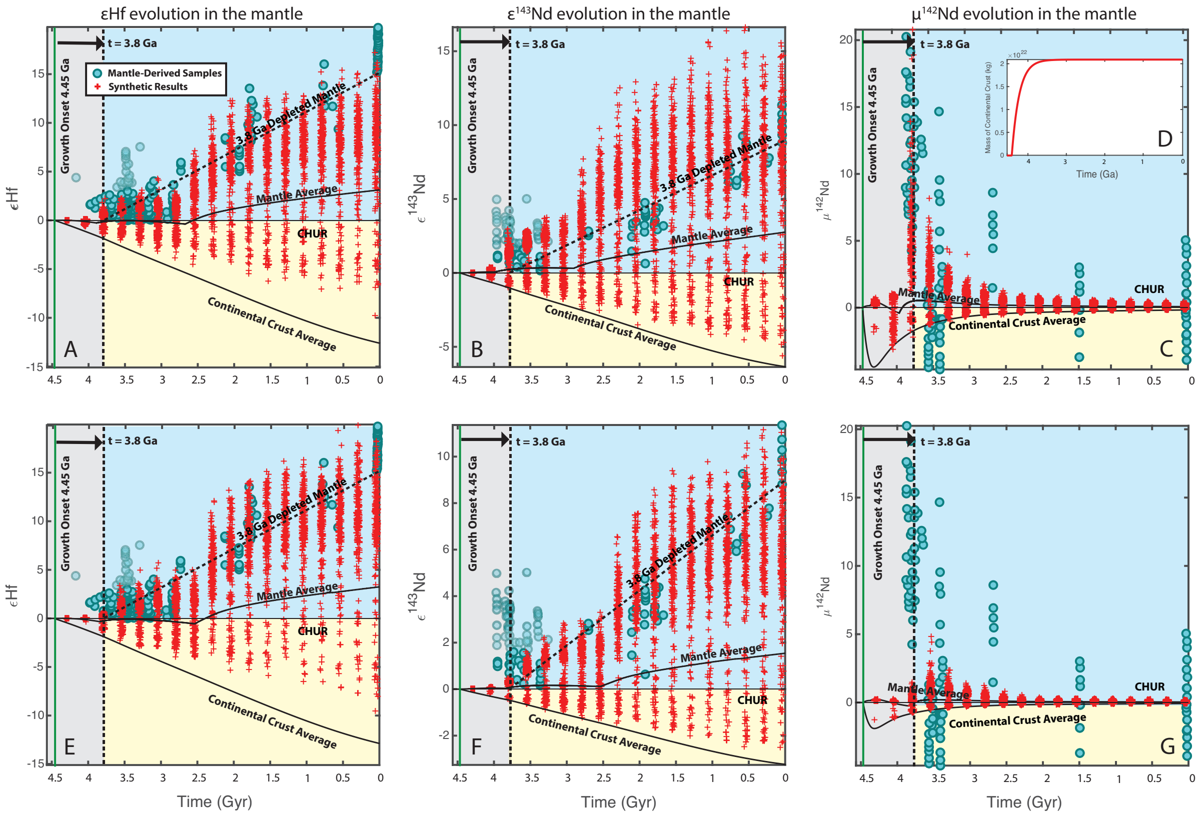

Fig. S2. The evolution of εHf, ε^{143}Nd, and μ^{142}Nd in the depleted mantle during continental formation, with a homogenous post-magma-ocean primitive mantle. The isotopic evolution of the depleted mantle with Nd being **(A-C)** much more incompatible ($D_{\mathrm{Sm}} = 0.0353$, $D_{\mathrm{Nd}} = 0.0268$) and **(E-G)** moderately more incompatible than Sm ($D_{\mathrm{Sm}} = 0.0353$, $D_{\mathrm{Nd}} = 0.0320$). **(D)** Net continental growth since 4.45 Ga. The observations of εHf, ε^{143}Nd, and μ^{142}Nd are shown in blue dots (*10, 15*, *16*, *29*, *31-35*, *37*, *38, 39*, *69-83,* and refs therein) and our modeling results are in red crosses. As the early strongly positive ε^{143}Nd signals may be subject to post-crystallization events (e.g., *18, 30, 31*) and the strongly positive εHf signals exist only in detrital zircons but not in magmatic zircons (*10*), we use a lighter shade for these observations. The vertical green lines at 4.45 Ga mark the onset of continental growth. The vertical dashed lines represent the timing of the earliest mantle depleted signals. The dashed lines labeled "3.8 Ga Depleted Mantle" represents the hypothetical evolution of depleted mantle when continental crust extracted at 3.8 Ga. The modeled isotopic evolutions of the average mantle and continental crust are also shown in solid curves. The blue and yellow backgrounds denote the domains in which our modeling results show significant positive and negative isotopic signals, respectively; whereas the grey background denotes the domain with no significant signals.

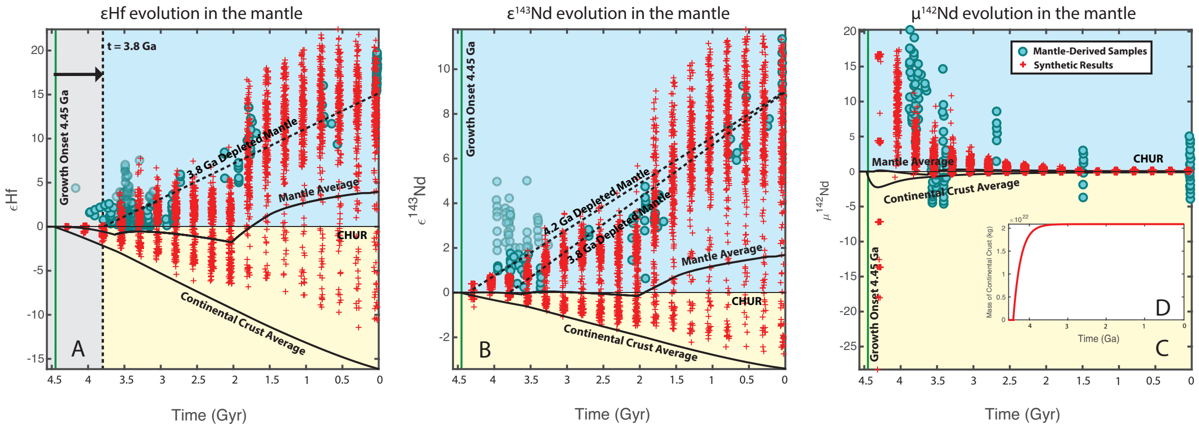

Fig. S3. Same as Fig. S2, but for the case of a heterogeneous post-magma-ocean primitive mantle. **(A-C)** The isotopic evolution of the depleted mantle with **(D)** rapid continental growth since 4.45 Ga.

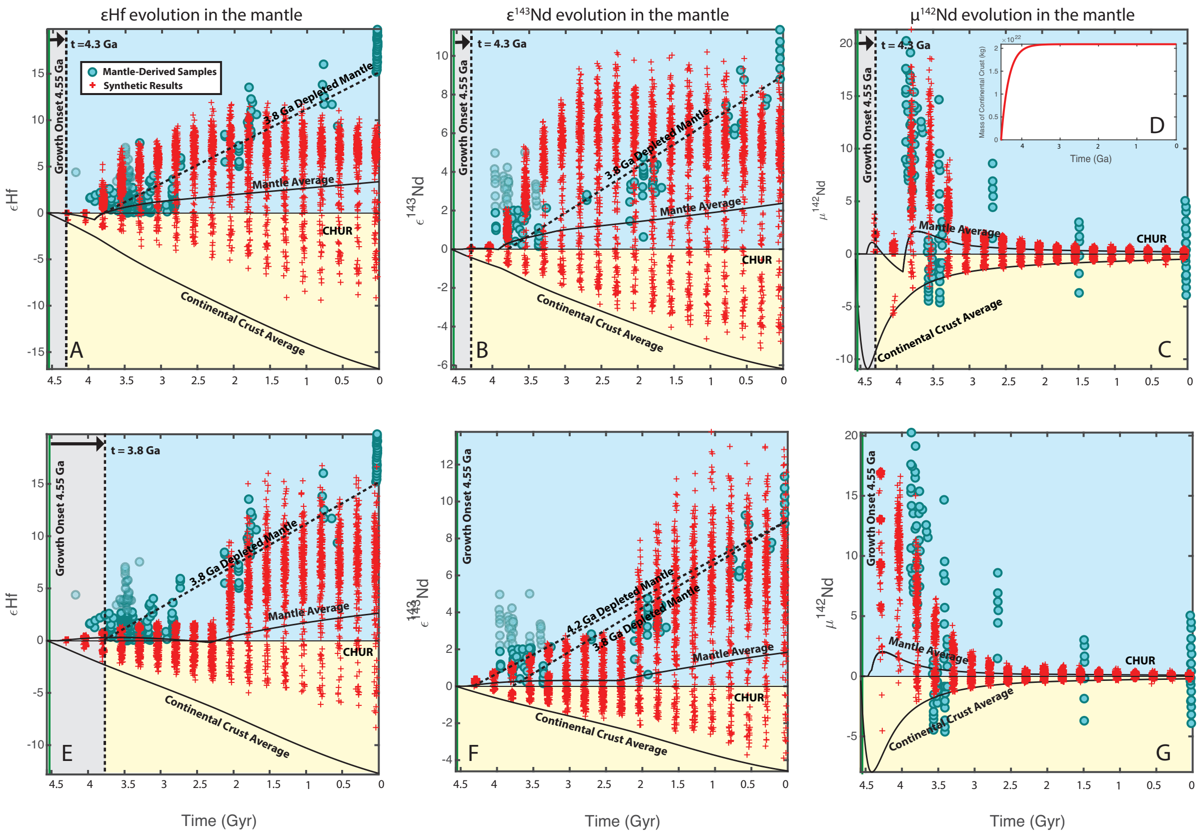

Fig. S4. Same as Fig. S2, but with mantle stretching rate being independent with volatile content. The chemical evolution in the depleted mantle with a **(A-C)** homogenous and **(E-G)** heterogenous primitive mantle after magma ocean solidification, during **(D)** rapid growth of continental crust since 4.55 Ga.

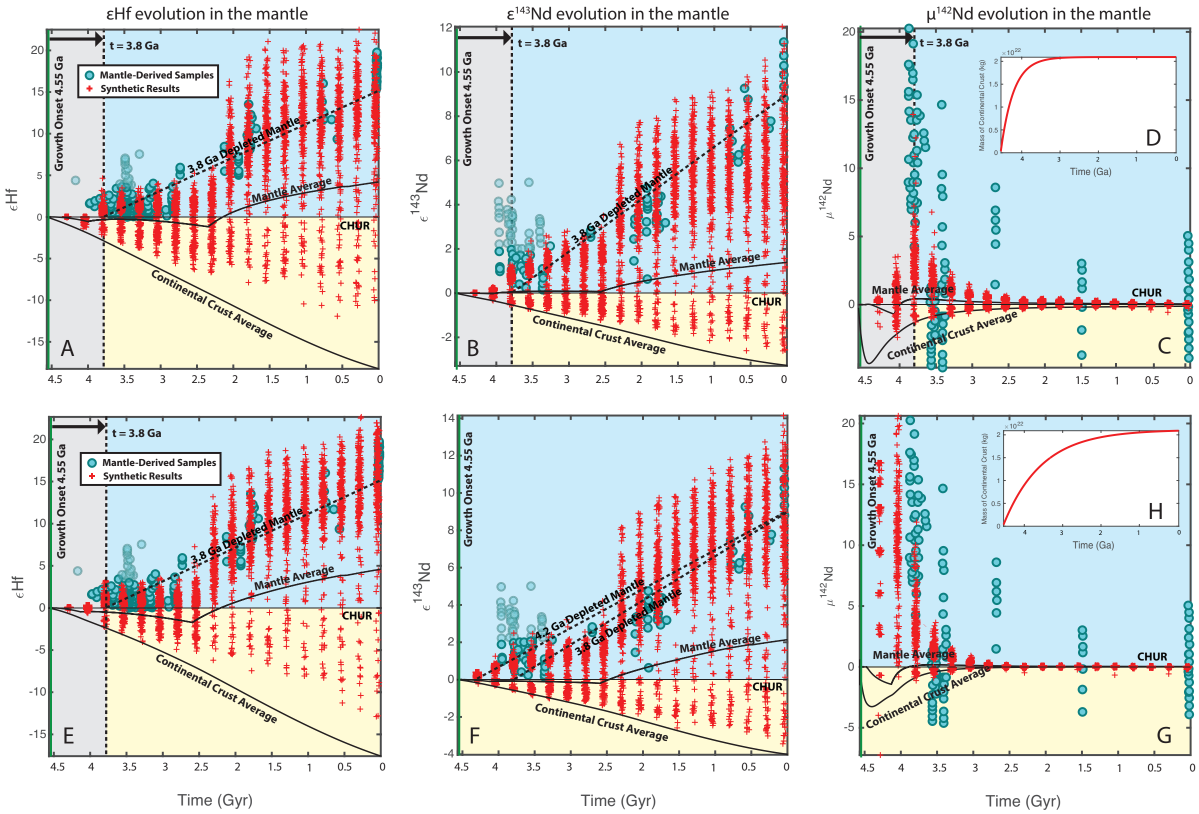

Fig. S5. Same as Fig. S2, but with early gradual growth of the continental crust. The chemical evolution in the depleted mantle with a **(A-C)** homogenous and **(E-G)** heterogenous primitive mantle after magma ocean solidification, during **(D)** moderately gradual growth and (**H)** gradual growth of continental crust since 4.55 Ga.
